# Clinicopathological profile of basal cell carcinoma: a retrospective study from Limpopo province, South Africa

**DOI:** 10.1007/s10238-025-01822-9

**Published:** 2025-08-06

**Authors:** Manenzhe Radzilani, J. McCusker Powell Casey, N. Mhlongo Lucia Naddy

**Affiliations:** 1 Pathology Division, National Institute for Occupational Health, National Health Laboratory Service, Johannesburg, South Africa; 2 School of Pathology, Faculty of Health Sciences, University of the Witwatersrand, Johannesburg, South Africa; 3 Department of Cellular Pathology, East Kent Hospitals University NHS Foundation, Ashford, Kent, UK; 4 School of Medicine, Faculty of Health Sciences, Department of Anatomical Pathology, Sefako Makgatho Health Sciences University, Molotlegi Road, Ga-Rankuwa, Pretoria, 0208 South Africa

**Keywords:** Basal cell carcinoma, Non-melanoma skin cancer, Limpopo Province, Skin tumours, Nodular BCC

## Abstract

Basal cell carcinoma is a non-melanoma skin cancer arising from basal epidermal keratinocytes. Its incidence is on the rise in the African continent and has been reported as the common skin malignancy worldwide, with a male predominance. The BCC subtypes are divided into low- and high-risk types based on prognostic characteristics. This study aimed to describe the demographic and clinicopathological characteristics of basal cell carcinoma cases from a predominantly native African population.A retrospective descriptive case series reviewed histopathological reports and archival slides of 86 basal cell carcinoma cases diagnosed at the National Institute for Occupational Health (NIOH), Pathology Division, from 1 January 2017 to 31 June 2021. Data were retrieved from the laboratory information system, captured into a Microsoft Excel spreadsheet and then analysed using STATA v.18.The mean age for the 86 study cases was 61 years, with a higher prevalence of BCC in females (64%). Most BCC lesions, 46 (53.5%), were located at the head and neck region. The low-risk nodular (45.4%; *n* = 39), the high-risk infiltrative (6.0%; *n* = 6) and basosquamous (5.8%; *n* = 5) were the most diagnosed BCC histopathological subtypes. Solar damage was recorded in (53.5%; *n* = 46) cases. Solar elastosis was associated with BCC subtype (odds ratio, 2.56; 95% CI, 1.0–6.3; *P* = 0.041).Basal cell carcinoma was more common in African females in their sixth decade. Age and nodular histopathological subtypes are consistent with most previous studies. These study findings highlight the growing incidence of BCC in the African population.

## Introduction

Basal cell carcinoma (BCC) manifests as slow-growing tumours with non-aggressive clinical behaviour [1]. The incidence of skin cancer, particularly basal cell carcinoma (BCC), is on the rise globally, posing significant health and financial burdens [1, 2]. Although BCC generally has a low mortality rate, its increasing prevalence remains a significant concern [2–4]. Among Caucasians, BCC is the most common type of skin cancer, occurring four to five times more frequently than squamous cell carcinoma (SCC) [1, 2, 5, 6]. Earlier studies reported predominance in males and rarity among individuals of African descent [5, 6]. BCC typically presents in regions with less sunlight in adults aged sixty or older [2].

South African studies reported various results in population of the various racial and ethnic groups, although black South Africans were not analysed separately [4, 7, 8]. A Western Cape study reported 85.9% in whites, mixed ancestry at 12.1%, and black Africans and Indians were 0.7% [4]. In Northern Cape, a study conducted on cutaneous malignancies reported BCC in 4.0% of blacks, 13% coloured, 0.3% Indians and 83.7% whites [7]. A study in Johannesburg reported BCC in 97.6% of patients with light skin [8].

Basal cell carcinoma (BCC) develops due to genetic, environmental, and individual factors [9]. The development of keratinocytic cancers has been linked to human immunodeficiency virus (HIV) in epidemic areas and ultraviolet radiation (UVR), depending on the geographic location [2, 6]. Immunocompromised individuals are also at heightened risk: South Africa is a Human immunodeficiency virus (HIV) epidemic country [1, 4, 6]. The abnormal activation of the Hedgehog signalling pathway is central to BCC, and it also interacts with other cancer-related pathways like EGFR, TGF-β, PI3K, NF-κB and aPKC, which play a role in the tumour’s progression [10, 11].

The head and face areas are most exposed to UV rays and are the most common anatomical site for BCC [1, 12]. Less often, lesions occur on the neck, trunk and proximal extremities and rarely involve the lower leg [3]. Clusters of basal cells’ characteristic of basal cell carcinoma (BCC) are identified by their scant cytoplasm and large, dark nuclei, set within a fibromyxoid stroma that contains retraction spaces [10, 13, 14]. Increased blood vessel formation around the tumour often indicates aggressive behaviour [10, 13]. The National Comprehensive Cancer Network (NCCN) has categorised the BCC subtypes based on their recurrence risk and clinical and pathological characteristics [13]. The low-risk types include nodular, superficial, pigmented, infundibulocystic, and fibroepithelial BCCs, while high-risk types include micronodular, infiltrating, sclerosing/morphoeic, basosquamous, and BCCs with sarcomatoid differentiation [13–15].

Nodular BCCs have large nests of basaloid cells with central disorganisation and peripheral palisading. Superficial BCCs appear as small islands of cells in the upper dermis, often with an inflammatory infiltrate [15–17]. Micronodular BCCs feature small, deeply infiltrative nodules, while infiltrating BCCs present with thin nests that penetrate deeply [17]. Sclerosing BCCs have very thin strands of cells within a dense collagenous stroma [15, 17]. Basosquamous carcinoma combines features of BCC and squamous cell carcinoma [15, 16]. Pigmented BCCs contain melanin due to increased melanocytes, and BCCs with sarcomatoid differentiation exhibit malignant basaloid cells in a sarcomatous stroma [16, 17]. Nodular and superficial subtypes are reported to be the most frequent and typically found in the head, neck and trunk areas [2].

The prevalence and clinicopathological characteristics of BCC and most skin cancers have been extensively studied in the USA, Australia and European countries. Given BCC’s rising prevalence, understanding its demographics and histopathology is crucial for treatment management and disease prevention. While many Western countries have studied skin tumour trends, this study aims to describe BCC demographics and histopathological characteristics in the African population from Limpopo province, South Africa. The findings will raise community awareness, aid healthcare providers in early detection, and improve patient management strategies.

## Materials and methods

### Study design, setting and sample

This retrospective descriptive case-series study was conducted on 86 (n = 86) cases of BCC diagnosed at the National Institute for Occupational Health (NIOH), Pathology division. The study sample consisted of all cases with BCC from hospitals across Limpopo province submitted to NIOH from 1 January 2017 to 30 June 2021. All cases with a histopathological diagnosis of BCC were included. Any other types of skin cancers were excluded. The Academic Affairs granted permission to conduct the study, Research and Quality Assurance Department of the National Health Laboratory Services (NHLS), reference number PR2119074. Ethical clearance was obtained from the Human Research Ethics Committee (HREC) at the University of Witwatersrand, certificate number M210967.

### Data collection

Systemised Nomenclature of Medicine (SNOMED) codes for basal cell carcinoma (M-80903, M-80973, M-80933) were used to conduct the search, and cases from 1 January 2017 to 30 June 2021 were retrieved from the Trakcare NHLS database. Demographic data (age at diagnosis, gender, ethnic group, albinism), clinical data (anatomical site of lesion and lesion size) and histopathological data (histopathological subtype, solar damage or actinic keratosis and tumour invasion) were recorded from histopathology reports obtained from the NHLS central data warehouse, confirmation and missing information was checked on the NHLS Trakcare laboratory information system. Cases with missing data that were crucial for this study were excluded from the analysis.

Solar damage/actinic keratosis was recorded according to grades. The presence of perineural invasion was also recorded. No sampling was done as this was a retrospective case-series review study. The case slides of the cases were retrieved and reviewed by experienced pathologists to confirm the initial diagnosis and for grading solar elastosis. To ensure the reliability and validity of the results, a third pathologist was invited to analyse the cases where there was no agreement, and the case discussions were done on cases that were difficult to diagnose independently.

The data received from the NHLS contain patient identifiers to enable the researcher to retrieve the case slides and blocks. The initial datasheet contained identifiers that enabled the researcher to analyse the race/ethnicity of the cases as well as retrieve the case slides. Patient confidentiality was always maintained. Reports retrieved were identified using only their allocated case numbers, and all samples were assigned research numbers prior to further data analysis and presentation. Data from the enrolled cases were captured on an Excel spreadsheet and stored in a password-protected computer and on the departmental external hard drive in a password-protected folder.

### Data analysis

The histopathological analysis and diagnosis of the cases were based on the World Health Organisation (WHO) classification for skin tumours and the National Comprehensive Cancer Network (NCCN) BCC guidelines criteria. Statistical analysis was done using Stata 16.1/MP (StataCorp, College Station, Texas, USA). Shapiro–Wilk test and Q–Q probability plots were used to assess the data’s distribution, skewness and kurtosis. Chi-square was used for categorical data. The results were summarised and presented in tables as count (percentages) for categorical data and median (25–75th percentile) for not normally distributed continuous data. A p-value of less than or equal to 0.05 was considered statistically significant.

A binary and multivariable logistic regression analysis was conducted to examine the influence of various clinical and pathological characteristics on gender, which served as the dependent variable in this study. A p-value of less than 0.05 was considered statistically significant for all analyses. The results of the logistic regression analyses were presented as odds ratios (ORs) accompanied by 95% confidence intervals (CIs), allowing for interpretation of the likelihood of being male or female based on the clinical and pathological characteristics.

## Results

### Demographic characteristics

**Fig. 1:**
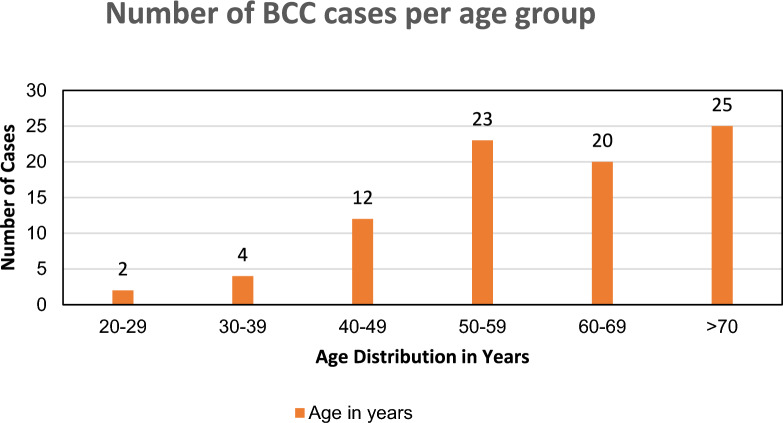
The age distribution of cases with BCC. Most BCC cases were diagnosed after the fifth decade (79.1%; *n* = 68)

A total of 86 cases with a reported diagnosis of BCC were identified. The age of the patients ranged from 23 to 89 years, with an overall mean age of 61 ± 14.22 years. The mean age for females was 62.2 and 58.9 for males; males were younger with a statistically insignificant *p*-value of 0.302. Figure 1 illustrates the age distribution for the study population. The higher percentage of the study population was of African origin; 59.3% and 40.7% were not indicated, though the family names were all African, with a female predominance (64%) and 36% male. The BCC cases increased with age, reaching the peak at > 70 years (Fig. 1).

### Clinicopathological characteristics

A total of 45 (52.3%) BCC lesions were reported in the head and neck area, 19 (22.1%) were in the trunk area and 11 (12.8%) were on the limbs. The lesion size was not reported on 51 (59.3%), 12 (14%) patients had a lesion size of less than 10 mm, 5 (5.8%) patients had a lesion size ranging from 10 to 20 mm and 13 (15.1%) patients recorded a lesion size of greater than 20 mm (Table 1). Perineural invasion was only recorded in three cases (3.5%) (Table 1).

**Table 1:** Demographic and clinical features of basal cell carcinoma cases

Variables	All, n (%)	Female, n (%)	Male, n (%)
*Anatomical area*			
Head/neck area	45 (54.2)	29 (55.8)	16 (51.6)
Head/neck/limbs/trunk	8 (9.6)	5 (9.6)	3 (9.7)
Limbs	11 (13.3)	6 (11.5)	5 (16.1)
Trunk	19 (22.9)	12 (23.1)	7 (22.6)
*Size of lesions*			
< 10 mm	12 (14.0)	7 (12.7)	5 (16.1)
10–20 mm	12 (14.0)	10 (18.2)	2 (6.5)
> 20 mm	11 (12.8)	8 (14.6)	3 (9.7)
Not stated	51 (59.3)	30 (54.6)	21 (67.7)
Invasion No	35 (40.7)	23 (41.8)	12 (38.7)
Yes	42 (48.8)	25 (45.5)	17 (54.8)
Unknown	9 (10.5)	7 (12.7)	2 (6.5)
*Ulceration*			
No	43 (50.0)	26 (47.3)	17 (54.8)
Yes	34 (39.5)	22 (40.0)	12 (38.7)
Unknown	9 (10.5)	7 (12.7)	2 (6.5)
*Solar damage/Elastosis Grade*			
Absent	40 (46.5)	21 (38.2)	19 (61.3)
1	6 (7.0)	5 (9.1)	1 (3.2)
2	13 (15.3)	12 (21.8)	1 (3.2)
3	27 (31.4)	17 (30.9)	10 (32.3)

*n = number of cases

**Fig. 2:**
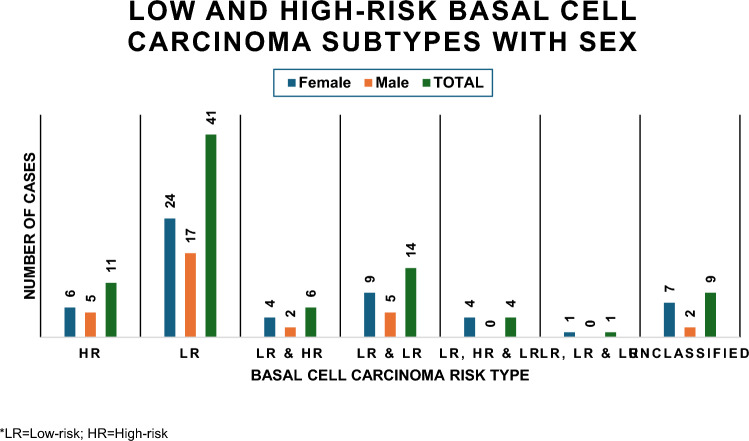
The low-risk basal cell carcinoma subtypes were most common in both males and females, and cases diagnosed with more than two subtypes were diagnosed in females

Table 2 and Fig. 2 represent the distribution of the histopathological grades of the cases. The most common histopathological subtype was the low-risk nodular subtype with 39 (45.4%) cases (Table 2). Most of the reported cases were low-risk (47.7%; *n* = 41) or a mixture of low-risk subtypes (16.3%; *n* = 16.3%) (Fig. 2).

**Table 2:** Distribution of the histopathological subtypes of basal cell carcinoma cases

Basal cell carcinoma subtypes	Number	Percentage
*High-risk*		
Basosquamous	5	5.8
Infiltrative	6	6.9
*Low-risk*		
Nodular	39	45.3
Superficial	2	2.3
*Low-risk & High-risk*		
Nodular and Infiltrative	5	5.8
Nodular and Morphoeic	1	1.2
*Low-risk & Low-risk*		
Nodular and Keratotic	8	9.3
Nodular and Superficial	5	5.8
Pigmented and Nodular	1	1.2
*Low-risk, High-risk & Low-risk*		
Nodular, Superficial and Infiltrative	1	1.2
Nodular, Nodular and Infiltrative	1	1.2
Nodular, Keratotic and Basosquamous	1	1.2
Nodular, Keratotic and Infiltrative	1	1.2
*Three low-risk*		
Superficial, Keratotic and Nodular	1	1.2
Unclassified	9	10.4
Total	86	100

Table 2 The distribution of the basal cell carcinoma histopathological subtypes. The low-risk nodular subtype was the most common.

### Albinism

Albinism was reported in only 16.5% of the cases. Table 3 illustrates the relationship between clinicopathological characteristics and BCC cases associated with albinism. The association between albinism and age was statistically significant, with albinism patients younger than the other patients (*p* < 0.001; mean age 45.6 ± 10.3 versus 64.1 ± 12.9). The association between albinism and site was significant at *p* = 0.027, with more cases reported in multiple areas (head, neck, limbs and trunk) and the trunk region at 28.6%.

**Table 3:** Association of clinicopathological features with albinism BCC cases

Variables	All (*n* = 86)	Albinism absent, 72 (83.7)	Albinism Present, 14 (16.3)	*P*-value
Age, (*n* = 86)	61.08 ± 14.3	64.1 ± 12.9	45.6 ± 10.3	< 0.001
Anatomical area (*n* = 82)				
Head/neck Area	45 (54.9)	39 (57.4)	6 (42.9)	
Head/Neck/Limbs/Trunk	8 (9.8)	4 (5.9)	4 (28.6)	
Limbs	11 (13.4)	11 (16.2)	0 (0.0)	
Trunk	18 (21.9)	14 (20.6)	4 (28.6)	0.027
Subtypes (*n* = 86)				
Basosquamous	5 (5.9)	4 (5.6)	1 (7.1)	
Nodular	39 (45.9)	32 (45.1)	7 (50.0)	
Infiltrative	6 (7.1)	4 (5.6)	2 (14.3)	
Superficial	2 (2.4)	2 (2.8)	0 (0.0)	
Combined	24 (28.2)	20 (28.2)	4 (28.6)	
Unknown	9 (10.6)	9 (12.7)	0 (0.0)	0.621
Solar damage (*n* = 86)				
Absent	40 (46.5)	37 (51.4)	4 (28.6)	
Present	46 (53.5)	35 (48.6)	10 (71.4)	0.129

*n = number of cases

The relationship between albinism and histopathological subtypes did not demonstrate statistical significance (*p* = 0.621); however, the nodular and combined subtypes were the most frequently observed. Although the association between albinism and solar damage was not statistically significant (*p* = 0.129), solar elastosis was present in the majority of cases.

### Association of clinicopathological and demographic data

**Fig. 3:**
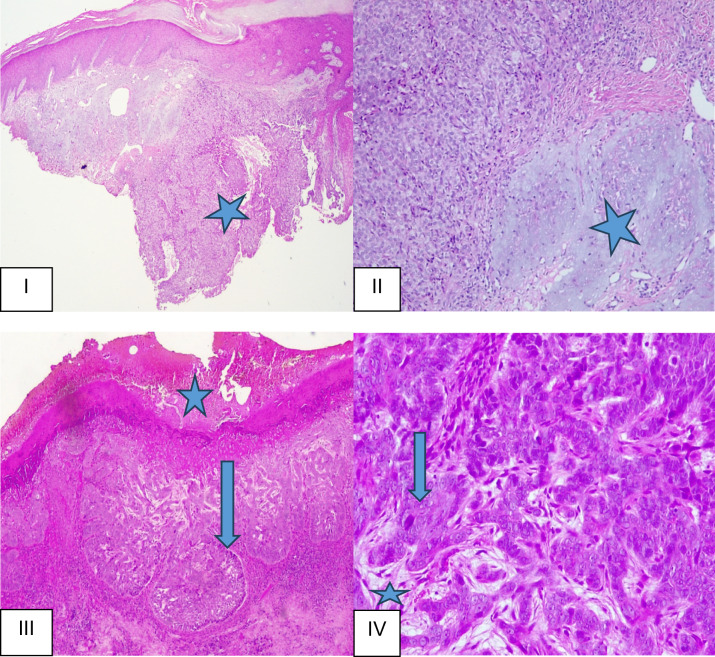
Haematoxylin and eosin micrographs of selected BCC study cases. Image I: This low-power view demonstrates skin tissue with the epidermis and dermis. There is invasive nodular BCC and severe actinic damage within the dermis (grade 3). (star-nodular BCC) (H&Ex25). Image II: This high-power view shows extensive actinic damage. (H&Ex200). Image III: This low-power view demonstrates skin tissue with epidermal ulceration (star) and tumour nests with peripheral palisading (arrow). (H&Ex50). Image IV: This high-power view demonstrates basaloid tumour cells in the background of myxoid stroma (star). The tumour cells have basaloid cytoplasm, the nuclei are pleomorphic with variable prominence of nucleoli, and some nuclei are hyperchromatic (arrow). (H&Ex200)

Table 4 presents the results of univariate and multivariate logistic regression analyses, with gender serving as the dependent variable and various clinical and pathological characteristics as independent variables (Fig. 3). In the univariate logistic regression model, only solar damage/elastosis grade was significant at *p* < 0.05, while in the multivariate none of the variables were significant. In the univariate analysis, females were 2 times more likely to present with solar damage, and 10 times more likely to have solar elastosis grade 2.

**Table 4:** Predictors of clinical/pathological features with sex as the outcome variable

Variables	Univariate logistic regression		Multivariate logistic regression	
	OR (95%CI)	*p*-value	OR (95%CI)	*p*-value
*Anatomical area*				
Head/neck Area	Ref			
Head/Neck/Limbs/Trunk	0.92(0.2;4.3)	0.916		
Limbs	0.66(0.2;2.5)	0.545		
Trunk	0.94(0.3;2.9)	0.922		
*Size of lesions*				
< 10 mm	Ref			
10–20 mm	1.50(0.3;8.8)	0.654		
> 20 mm	1.33(0.2;7.9)	0.753		
*Invasion*				
No	Ref			
Yes	0.77(0.3;1.9)	0.577		
*Ulceration*				
No	Ref			
Yes	1.20(0.5;3.0)	0.703		
*Additional pathology*				
None	Ref			
Perineural invasion	1.13(0.1;13.0)	0.921		
*Solar damage*				
Absent	Ref		Ref	
Present	2.56(1.0;6.3)	0.041	1.53(0.6;4.2)	0.398
*Solar damage/Elastosis Grade*				
Absent	Ref		Ref	
1	3.61(0.4;35.3)	0.268	2.35(0.2;24.1)	0.471
2	10.86(1.3;21.6)	0.028	7.06(0.8;62.7)	0.080
3	1.53(0.6;4.2)	0.398		
*Subtypes*				
Basosquamous	Ref			
Nodular	0.35(0.04;3.5)	0.379		
Infiltrative	0.13(0.01;1.9)	0.141		
Superficial	0.25(0.01;8.6)	0.442		
Combined	0.64(0.1;6.8)	0.714		

#combined = Nodular and Keratotic, nodular and morphoeic, nodular and superficial, nodular and pigmented, nodular, keratotic and basosquamous, nodular, keratotic and infiltrative, nodular, nodular and infiltrative, nodular, superficial and infiltrative, superficial, keratotic and nodular

The association between age and histopathological subtypes was statistically insignificant with all subtypes (Table 4).

## Discussion

The global incidence of skin cancers has steadily increased over recent decades, with a notably sharp rise among young women [18]. Basal cell carcinoma is a common skin malignancy whose incidence has been reported to be on the rise within the African continent. The HIV epidemic and warmer climatic conditions within the African region might be central to the aetiopathogenesis of BCC in South Africa (SA) [9]. America, Europe and Australia have conducted various studies on BCC, yet the data on BCC within the African continent remain limited. The diverse racial and tribal groups in SA also contribute to the different susceptibility within the different population groups. The case series aimed to report the trends of BCC in a black-populated Limpopo province.

The province has the majority of its population as Xitsonga, Tshivenda and Sepedi native tribes, with few other tribes; the data were collected within public government Hospitals, which serve mainly the low-income class, and no data were received from private hospitals/clinics. The BCC cases were reported more frequently in females (64%), a distinctive finding as studies from the USA, India and other provinces in South Africa reported a male predominance [2, 4, 7, 9, 19, 20]. This study had a cohort of 86 cases from hospitals across the Limpopo province, of which 64% were female and 36% male, with solar elastosis reported on 53.5% of the entire cohort. Earlier studies linked male BCC predominance to prolonged sun and chemical exposure [9, 21, 22].

Literature has linked the higher BCC rates in younger women to lifestyle factors such as indoor tanning and the use of other cosmetics [18, 23, 24]. There are minimal data on skin cancer in women of colour, making it hard to determine true incidence and mortality as well as its aetiopathogenesis [18, 23]. Increased awareness and understanding are needed to address skin cancer in this population [24]. As many women of colour experience changing socioeconomic conditions, education on BCC would reduce delayed care [18, 23]. The current study was retrospective; the exact link between socioeconomic status and cosmetic usage could not be determined.

Contrary to the most reported literature, a Singapore study reported a 1:1 male-to-female ratio in BCC cases. The study included Chinese, Malays and Indians, and the Chinese were at an increased risk of developing skin cancer [25]. Limpopo province is a region of South Africa well known for farming, agricultural activities, and very warm climate conditions. Studies have reported a higher number of females employed in the farming sector; hence, prolonged occupational (UVR) ultraviolet ray exposure might contribute to increased skin tumours in females in this population cohort [26]. A study conducted in 2021 in China highlighted an increasing number of BCC cases in females, even though males are still twice as likely to develop BCC [27]. A study done in 2021 in Europe found BCC to be more associated with occupational UVR exposure than SCC [28]. These studies also reported that individuals working in the agriculture and farming sectors are more at risk, as most activities are outdoors [28, 29].

Most of the cases in the current study had more individuals aged 50 years and older, a finding in keeping with that of various studies, reporting BCC to be more common in the sixth decade of life [2, 25]. This study had a mean age of 58 years for males and 62 years for females, which supports the mean age reported by Hakverdi et al., in India; the mean age was 64.1 in males and 59.3 in females [2, 25]. Studies in North America and India reported differently and found the mean age in BCC patients to be 40 years or in the fourth decade of life, respectively [11, 30].

The study cohort had (58.1%) individuals of African ethnicity, with the remainder of ethnicity not indicated, though the family names were of African ancestry. This is an uncommon finding compared to most literature that reported BCC to be less common in people of African origin, dark skin or non-Caucasians [1, 25, 30–32]. Another earlier study suggested that UVR occupational exposure is only associated with skin tumours in Caucasian individuals residing in certain geographical areas [27].

The study cohort reported very few cases of albinism. An African-based study found albinism to be a risk factor for developing skin cancer due to the lack of melanin in individuals with albinism and extreme exposure to UVR [33]. Albinism in African individuals was also reported to increase the risk of developing skin cancer by 1000-fold [33]. Due to the few cases of albinism in this study, the association between albinism and BCC could not be determined. However, most of the cases with albinism had multiple lesions and multiple subtypes. Furthermore, the cases with albinism showed a similar trend of histopathological subtypes to the other study sample.

The study reported 50% (*n* = 45) of BCC arising from the neck and head areas. The study findings are similar to those of various studies that reported BCC mainly affecting chronically sun-exposed skin areas of the head and neck regions [1, 2, 4, 29, 34]. There were no documented cases of recurrent BCC in this study population. Nodular subtype BCC was recorded to be the most frequent BCC histopathological subtype, which is in keeping with studies done in France, India, the USA and Africa, Western Cape, which reported the nodular BCC subtype to be the most common and usually occurs on the head and neck areas [1, 2, 4, 11, 33]. About 8.1% (*n* = 7) of the study population presented with more than one tumour; of these cases, 57.1% (*n* = 4) had albinism; Hakverdi et al., in 2011, reported 12 cases with more than one tumour [2].

Infiltrating types were reported to be 43.3% by Hakverdi et al. 2011 and 14.8% by Pampena et al. 2022, and more common in the neck and head region; this prevalence is much higher than that of the current study [20]. Niculet et al., 2023 reported an association between perineural invasion and chronic perineural inflammation to be linked to high-grade tumours of aggressive nature [34]. In the current study, perineural invasion was documented in 3.5% of the cases, which was too low a number to associate with specific subtypes.

While some research suggests that men are more prone to skin damage due to increased outdoor exposure without adequate sun protection [35], a binary logistic model from the present study indicated that women were 2.5 times more likely to exhibit solar damage. However, this finding was not statistically significant in the multivariate model. Females in these study were 10 times more likely to have Elastosis Grade 2 than males; however, this finding was not significant in the multivariate model, suggesting that other factors may play a more critical role. Furthermore, males with Elastosis Grade 2 might have been underrepresented in the study.

Most of the findings reported in the current study are in keeping with local and international studies, apart from gender and ethnicity, which were reported distinctively. These findings also indicate a need for further studies to be conducted in all geographic areas with different population groups to identify the risk groups and assist clinicians with accurate and timely patient care. The retrospective nature of the study was a limitation as some of the risk factors, like immunosuppression, radiation therapy, lifestyle factors, and socioeconomic status, could not be explored.

## Conclusion

The study found a higher frequency of BCC in females compared to males. Diagnosed patients were of African ethnicity in their sixth decade of life and older. The head and neck region were the most common anatomical site, and nodular BCC was the most frequent histological subtype. This study demonstrated that BCC is prevalent in the African native population of the Limpopo province. The clinicopathological characteristics observed in this population are similar to those observed in other local and international studies, except for the gender distribution. Excision with adequate margins is necessary for the infiltrating subtypes to minimise recurrence.

### Study limitations

This was a retrospective study that depended on the accuracy of information provided by clinicians as captured on the laboratory information system, and as such, there were missing data points. A prospective study will enable complete and accurate patient information collection, enabling the comparison of risk factors.

## Data Availability

Data to support the findings published on this study are available from the corresponding author LNM on request.
