# Correction: Clinicopathological profile of basal cell carcinoma: a retrospective study from Limpopo province, South Africa

**DOI:** 10.1007/s10238-026-02215-2

**Published:** 2026-06-19

**Authors:** Radzilani Manenzhe, Casey Powell McCusker, Lucia Naddy Mhlongo

**Affiliations:** 1 Pathology Division, National Institute for Occupational Health, National Health Laboratory Service, Johannesburg, South Africa; 2 School of Pathology, Faculty of Health Sciences, University of the Witwatersrand, Johannesburg, South Africa; 3 Department of Cellular Pathology, East Kent Hospitals University NHS Foundation, Ashford, Kent, UK; 4 School of Medicine, Faculty of Health Sciences, Department of Anatomical Pathology, Sefako Makgatho Health Sciences University, Molotlegi Road, Ga- Rankuwa, Pretoria, 0208 South Africa

**Clinical and Experimental Medicine (2025) 25:276**

10.1007/s10238-025-01822-9

In the original version of this article, the given and family names of the authors Radzilani Manenzhe, Casey Powell McCusker and Lucia Naddy Mhlongo were incorrectly structured:

The correct structure are as follows:

Given name: Radzilani

Family name: Manenzhe

Given name : Casey Powell

Family name : McCusker

Given name : Lucia Naddy

Family name : Mhlongo

